# Video Tutorials to Empower Caregivers of Ill Children and Reduce Health Care Utilization: A Randomized Clinical Trial

**DOI:** 10.1001/jamanetworkopen.2023.36836

**Published:** 2023-10-12

**Authors:** Liv Borch-Johnsen, Caroline Gren, Stine Lund, Fredrik Folke, Morten Schrøder, Marianne Sjølin Frederiksen, Freddy Lippert, Annette Kjær Ersbøll, Gorm Greisen, Dina Cortes

**Affiliations:** 1 Department of Pediatrics and Adolescent Medicine, Copenhagen University Hospital—Amager and Hvidovre, Copenhagen, Denmark; 2 Department of Clinical Medicine, University of Copenhagen, Copenhagen, Denmark; 3 Emergency Medical Services Capital Region, Denmark; 4 Hans Christian Andersens Childrens Hospital, Odense University Hospital, Odense, Denmark; 5 Department of Neonatology, Copenhagen University Hospital—Rigshospitalet, Copenhagen, Denmark; 6 Department of Cardiology, Copenhagen University Hospital—Herlev and Gentofte, Copenhagen, Denmark; 7 Department of Pediatrics and Adolescent Medicine, Copenhagen University Hospital—Rigshospitalet, Copenhagen, Denmark; 8 Department of Pediatrics and Adolescent Medicine, Copenhagen University Hospital—Herlev and Gentofte, Copenhagen, Denmark; 9 Falck Healthcare, Copenhagen, Denmark; 10 National Institute of Public Health, University of Southern Denmark, Copenhagen, Denmark

## Abstract

**Importance:**

Young children often fall ill, leading to concern among their caregivers and urgent contact with health care services.

**Objective:**

To assess the effectiveness and safety of video tutorials to empower caregivers in caring for acutely ill children.

**Design, Setting, and Participants:**

Caregivers calling the out-of-hours Medical Helpline 1813 (MH1813), Emergency Medical Services Capital Region, Denmark, and their children aged 0.5 to 11.9 years were randomized to video tutorials (intervention) or telephone triage by a nurse or physician (control) from October 2020 to December 2021 and followed up for 72 hours blinded to the intervention. Data were analyzed from March to July 2022.

**Intervention:**

The intervention group had the call disconnected before telephone triage and received video tutorials on managing common symptoms in acutely ill children and when to seek medical help. Caregivers could subsequently call MH1813 for telephone triage.

**Main outcomes and measures:**

The primary outcome was caregivers’ self-efficacy, reported in an electronic survey the following day. Secondary outcomes were satisfaction, child status, assessment by a general practitioner or physician at the hospital, telephone triage, and adverse events during the 72-hour follow-up period.

**Results:**

In total, 4686 caregivers and children were randomized to intervention (2307 participants) or control (2379 participants), with a median (IQR) child age of 2.3 (1.3-5.1) years and 53% male distribution in both groups (2493 participants). Significantly more caregivers in the intervention group reported high self-efficacy (80% vs 76%; crude odds ratio [OR], 1.30; 95% CI, 1.01-1.67; *P* = .04). The intervention group received fewer telephone triages during follow-up (887 vs 2374 in the control group). Intention-to-treat analysis showed no difference in secondary outcomes, but per-protocol subanalysis showed fewer hospital assessments when caregivers watched video tutorials (27% vs 35%; adjusted OR, 0.67; 95% CI, 0.55-0.82). Randomization to video tutorials did not increase adverse outcomes.

**Conclusions and relevance:**

In this randomized clinical trial, offering caregivers video tutorials significantly and safely increased self-efficacy and reduced use of telephone triage. Children had fewer hospital assessments when caregivers watched videos. This suggests a future potential of health care information to empower caregivers and reduce health care utilization.

**Trial Registration:**

ClinicalTrials.gov Identifier: NCT04301206

## Key Points

**Question:**

Can video tutorials empower caregivers when caring for acutely ill children at home?

**Findings:**

This randomized clinical trial included 4686 caregivers who had called a medical helpline regarding their children, with the intervention group receiving video tutorials and the control group receiving telephone triage by health care professionals (usual care). Significantly more caregivers randomized to video tutorials reported high self-efficacy compared with controls with no increase in adverse outcomes in children (ambulance admissions, ICU admissions, or death) during the 72-hour follow-up period.

**Meaning:**

These findings suggest video tutorials may effectively and safely increase empowerment to care for acutely ill children.

## Teaser

This randomized clinical trial evaluates the effectiveness and safety of video tutorials to empower caregivers in caring for acutely ill children in Denmark.

## Introduction

Children aged less than 3 years experience an average of 3 ill days per month.^1^ Although symptoms are usually mild and self-limiting, many caregivers seek urgent care due to concerns and overestimation of illness severity.^2,3,4^ This has led to an increase in urgent contacts to medical services,^5^ with hospital visits increasing in the United Kingdom since 1999.^6,7,8^ In the US, 22% of visits to emergency departments cared for children younger than 15 years.^9,10^ Furthermore, medical professionals classified fewer than half of the acute contacts as urgent.^9,10^ In the Capital Region of Denmark, approximately 25% of calls to the free out-of-hours service Medical Helpline 1813 (MH1813) concerned children, with 40% of these calls resulting in hospital referrals for further assessment.^11^

Caregivers often use online health information before seeking medical attention when a child falls ill,^12,13^ but readability and reliability limit the use of the existing information.^14,15^ Additionally, most health information focuses on the disease and medical treatment, rather than observed symptoms and methods to relieve them. The high number of urgent contacts regarding nonserious, self-limiting illnesses indicates that available health information may be inadequate. Strategies to empower parents in caring for children with mild symptoms and no need for treatment could improve health care utilization.^14,15,16,17,18,19^ Several approaches have been used, with varying results.^19,20,21,22^

In 2018, the World Health Organization recognized health literacy as a critical pillar in health promotion and addressed the potential need for digital media.^23^ We developed 9 short video tutorials, called Tips from Pediatricians,^24,25^ providing advice for home management and when to seek medical help on the most common symptoms in acutely ill children (fever, vomiting and diarrhea, stomach pain, breathing difficulties, sore throat, red eyes, earache, rash, and subsequently COVID-19).^1,9^ They were developed by a multidisciplinary group of experts on pediatric acute care from different parts of Denmark and evaluated by caregivers in focus groups.^24^

This randomized clinical trial aimed to assess the effectiveness of video tutorials to increase the self-efficacy of caregivers on how to care for acutely ill children and reduce health care utilization. The study compared an intervention group receiving video tutorials with a control group that received standard telephone triage by a nurse or physician. The hypothesis was that video tutorials would increase self-efficacy and reduce health care utilization without increasing adverse outcomes.

## Methods

### Study Design

In a 2-group, investigator-blinded, randomized clinical trial, we studied the effectiveness and safety of using video tutorials to empower caregivers who called a medical helpline regarding children with acute illness. The study was conducted from October 11, 2020, to December 13, 2021, and registered in the ClinicalTrials.gov database (see Trial Protocol in Supplement 1). This study followed the Consolidated Standards of Reporting Trials (CONSORT) reporting guideline.

The research ethics committee in the Capital Region, Denmark, deemed no approval necessary under Danish law. The study was registered and approved by the local data registration. Caregivers gave informed consent, with the right to withdraw from the study at any time, following the Danish Code of Conduct for Research Integrity and the Danish Data Protection Agency.^26^ The study adhered to the Declaration of Helsinki.^27^

MH1813, Copenhagen Emergency Medical Services, Denmark, is a free out-of-hours medical helpline that triages acute, non–life-threatening illnesses and injuries to all 11 hospitals in the Capital Region of Denmark. MH1813 serves 1.9 million citizens.^28^ Eligible participants were caregivers who called MH1813 regarding their child with acute illness aged 0.5 to 11.9 years. Exclusion criteria were calls regarding injuries, non-Danish speakers, declined participation, or inability to watch videos. Excluded calls underwent standard telephone triage. Callers to MH1813 provided the Civil Registration Number, a unique identifier that discloses age and gender, but not personal information such as socioeconomic status or ethnicity. Eligible callers were presented with voice-recorded information about the project and could press 2 to consent to participation (see Trial Protocol in Supplement 1). Those meeting exclusion criteria were instructed to press 3 for standard telephone triage, excluding them from the study. Participants were randomly assigned to intervention or control groups in a 1:1 ratio using a computer algorithm. Data were collected blinded, but participation blinding was not feasible due to the study setup.

### Study Intervention and Follow-Up

The intervention group had the call disconnected before telephone triage and received a link on their smartphone to video tutorials on the management of the most common symptoms in acutely ill children and when to seek medical help (eFigure in Supplement 2).^24,25^ The link was accessible through a personalized login for 72 hours to monitor viewership and prevent sharing with other households. The control group was directed to standard telephone triage with a nurse or physician (usual care). Repeated calls to the MH1813 72 hours after the initial call were directed to standard telephone triage.

Both groups received an electronic survey via text message the day following the initial call (see Trial Protocol in Supplement 1). We registered subsequent triage calls to the MH1813 (LogisCad patient data collection system) and hospital assessment 72 hours after the initial call (electronic medical record system Sundhedsplatformen/Epic). Hospital data were manually collected, reviewed within 4 to 6 days, and stored in a Research Electronic Data Capture (REDCap) system.

### Outcomes and Safety Measures

The primary outcome was high self-efficacy, defined a priori as caregivers reporting “to a high/very high degree” in 2 out of the 3 following items: “How well could you care for your sick child at home, after being in contact with the MH1813?”; “Do you know what to do, at home, if your child experiences similar symptoms again?”; and “Do you know when symptoms are severe and when you need to call your general practitioner/MH1813 if these symptoms reappear?” Secondary outcomes were the remaining items in the electronic survey (assessment by a general practitioner or physician at a hospital during the current illness period, satisfaction with MH1813, and status of the child) and 72-hour follow-up on telephone triage at MH1813, assessment in the hospital, and video tutorial viewership in the intervention group (see Trial Protocol in Supplement 1). The survey was developed by the research group with assistance from communication consultants to enhance clarity, comprehensibility, and response options. The survey items were pretested on a small group of Danish-speaking caregivers through interviews, and a final version was sent via text message to refine the survey and address any potential issues before trial.

Severe adverse events were defined as hospital admissions by ambulance, intensive care unit admissions, or death. A safety committee with pediatric experts not involved in this study received monthly unblinded hospital assessment reports and immediate notification of severe adverse events.

### Statistical Analysis

The sample size was based on the primary outcome, high self-efficacy, assuming 50% of caregivers reported high self-efficacy and a 40% response rate. Detecting a 5% increase required a total of 3300 completed surveys (80% power, 2-sided *P* < .05). This required a total of 8250 participants. A preplanned interim analysis at 400 completed surveys, to estimate the frequency of high self-efficacy and response rate, showed that 83% reported high self-efficacy and a 34% response rate. No other statistical analysis was conducted. To avoid ethically unjustified prolongation of the inclusion period, the sample size was recalculated to a total of 1568 completed surveys, which translated to 4611 participants.

Before the trial, a statistical analysis plan was developed with intention-to-treat (ITT) as the primary approach. Categorical outcomes were analyzed by proportional differences with Fischer exact test. For continuous and ordinal variables, the Mann-Whitney-Wilcoxon test was used. We calculated odds ratios (OR) and corresponding 95% CIs using unconditional logistic regression to determine the link between exposure and outcomes.

As a sensitivity measure, we conducted a per-protocol analysis, which included caregivers in the intervention group who viewed 1 or more video tutorials and caregivers in the control group who received standard telephone triage. Children with hospital-assessed injuries were excluded from both groups. The median child’s age deviated between the groups, and to address the potential confounding effect, outcomes were analyzed using a generalized linear model with a binomial distribution adjusted for the child’s age in tertiles with an equal distribution: 0.5 to 1.5, 1.6 to 3.9, and 4.0 to 11.9 years. Adjusted results are reported as ORs (aOR) with a corresponding 95% CIs.

All statistical analyses were performed after completed blinded data collection using RStudio statistical software, version 2022.07.01 (R Project for Statistical Computing). The threshold for statistical significance was set at 2-sided *P* < .05. Data were analyzed from March to July 2022.

## Results

**Figure 1.**
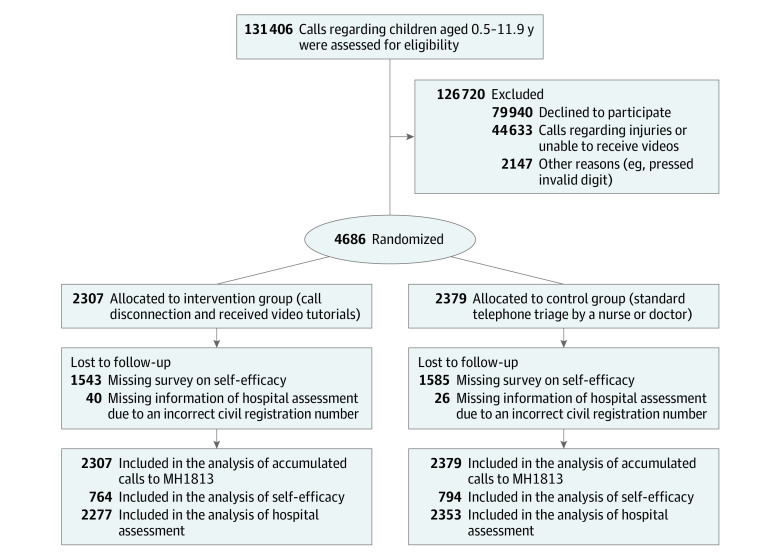
Flow Diagram of Participants

Among 131 406 eligible calls, 4686 were included in the trial (Figure 1). The remaining 126 720 calls were excluded for reasons including 79 940 declining to participate, 44 633 regarding injuries or inability to watch video tutorials, and 2147 being excluded for other reasons (Figure 1). The trial included 2307 in the intervention group and 2379 in the control group. The median (IQR) age of the children was 2.3 (1.3-5.1) years, with a 53% male distribution in both groups (2493 participants) (Table).

**Table.** Baseline Characteristics of Participants

Characteristic	Participants, No. (%)		
	All (N = 4686)	Intervention (n = 2307)	Control (n = 2379)
Child age, median (IQR), y	2.3 (1.3-5.1)	2.2 (1.2-5.1)	2.4 (1.3-5.1)
Child sex			
Male	2493 (53)	1227 (53)	1266 (53)
Female	2193 (47)	1080 (47)	1113 (47)
Phone number registered to			
Mother	2832 (60)	1367 (59)	1465 (61)
Father	879 (19)	433 (19)	446 (19)
Household	210 (5)	118 (5)	92 (4)
Not traceable	765 (16)	389 (17)	376 (16)

Children whose caregivers declined to participate had a median (IQR) age of 2.8 (1.4-6.3) years. In total, 34% of the children in both the control group (653 participants) and among those who declined to participate (24 012 participants) were referred to the hospital (eTable 1 in Supplement 2).

### Primary Outcome

**Figure 2.**
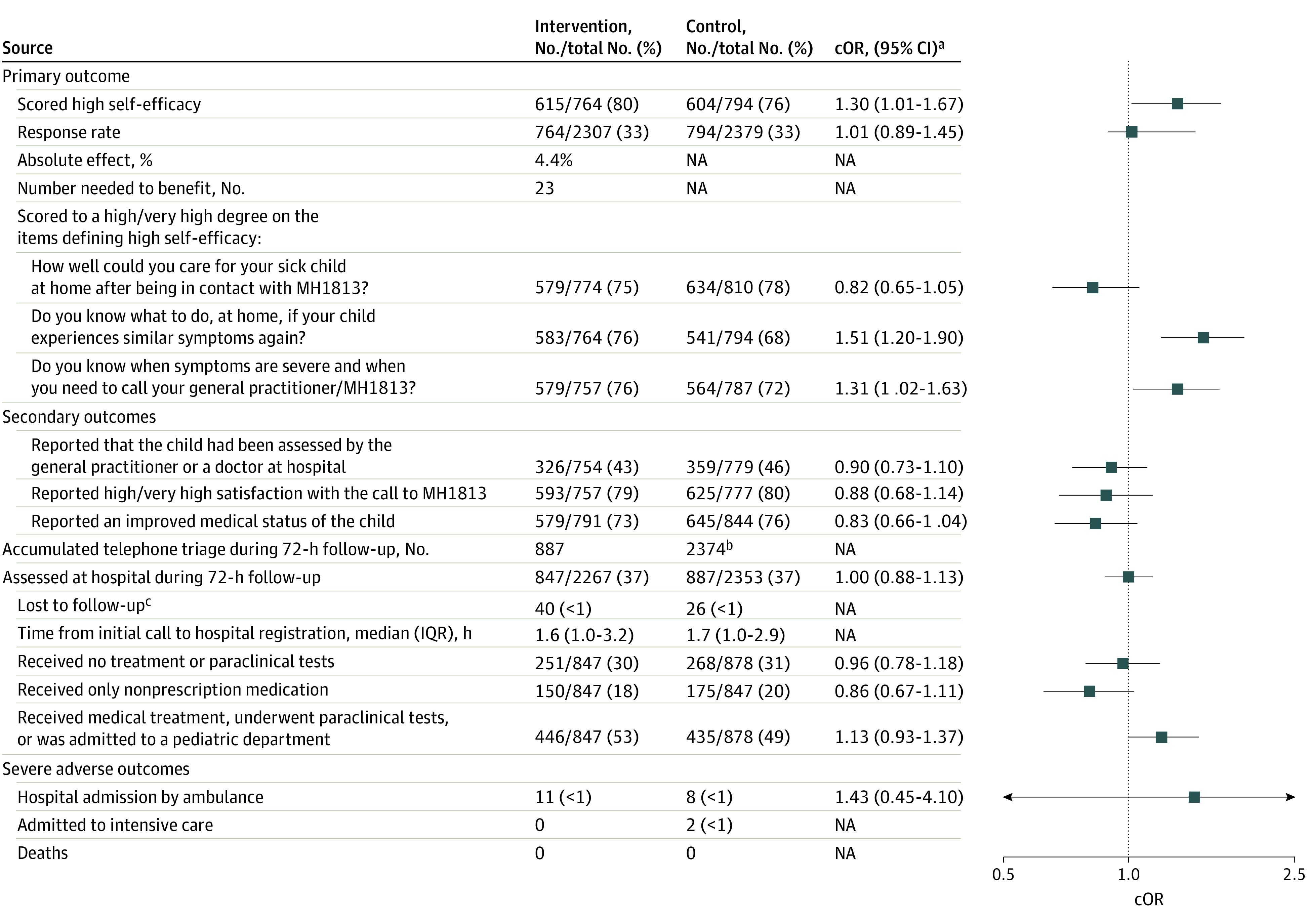
Intention-to-Treat Analysis: Primary, Secondary, and Adverse Outcomes cOR indicates crude odds ratio; MH1813, Medical Helpline 1813; NA, not applicable. ^a^Analyzed with Fisher exact, Rstudio Epitools package. ^b^Includes the 1920 telephone triage received during the initial call. ^c^Lost to follow-up was caused by incorrect civil registration number.

The response rate on the items defining self-efficacy was 33% (1558 of 4686 participants) with an equal distribution in both groups (Figure 2). Significantly more caregivers in the intervention group reported a high self-efficacy compared with the control group: 80% (615 of 764 participants) vs 76% (604 of 794 participants) (crude odds ratio [cOR], 1.30; 95% CI, 1.01-1.67; *P* = .04) (Figure 2).

An exploratory analysis of the items defining self-efficacy revealed that more caregivers in the intervention group reported “to a high degree/very high degree” to the following items: *“*Do you know what to do at home the next time your child experiences similar symptoms?” and “Do you know when symptoms are severe and when you need to call your general practitioner/MH1813 if these symptoms reappear?*”* (Figure 2). We found no difference between the groups regarding the item “How well could you care for your sick child at home, after being in contact with the MH1813?” (Figure 2). Details on survey responses are found in eTable 2, eTable 3, and eTable 4 in Supplement 2.

### Secondary Outcomes and Adverse Events

The caregivers and children in the intervention group received a total of 887 accumulated telephone triages (Figure 2), half of which were performed 15 minutes from the initial call (eTable 5 in Supplement 2). In comparison, 1920 caregivers in the control group received telephone triage during the initial call and a further 454 times during follow-up, resulting in a total of 2374 telephone triages (Figure 2).

In both groups, 37% of the children were assessed at the hospital during the 72-hour follow-up period (847 of 2267 in the intervention group vs 887 of 2353 in the control group). The median (IQR) time from the initial call to registration at the hospital was 1.6 (1.0-3.2) hours in the intervention group and 1.7 (1.0-2.9) hours in the control group (Figure 2). In total, 30% of the children in the intervention group (251 of 847 participants) vs 31% in the control group (268 of 878 participants) assessed at the hospital presented with mild symptoms and no need for treatment or paraclinical tests, and 18% (150 of 847 participants) vs 20% (175 of 878 participants) were only treated with nonprescription medicine in the intervention and control groups, respectively (Figure 2). The incidence of hospital admissions by ambulance was similar between the groups: 0.5% (11 of 2267 participants) vs 0.4% (8 of 2353 participants) (cOR 1.43; 95% CI, 0.45-5.10). No children in the intervention group required admission to intensive care, while 0.1% (2 of 2353 participants) of the children in the control group did (Figure 2). There was no indication to pause recruitment due to severe adverse events or caregiver withdrawals. Details on hospital assessments are found in eTable 6, Supplement 2.

**Figure 3.**
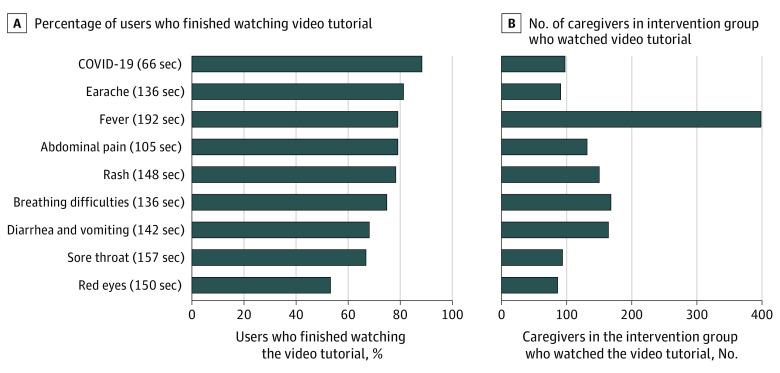
Video Tutorial Usage

The video tutorials were watched by 36% of the caregivers in the intervention group (831 of 2307 participants), who watched on average 1.7 videos. The video tutorial “Fever” was the most viewed (399 participants), followed by “Diarrhea and Vomiting” (162 participants) (Figure 3). “Red Eyes” had the lowest viewership (77 participants) (Figure 3). Most video tutorials were watched until the end with repeated sequences, except “Red Eyes,” which was watched until the end only 53% of the time (Figure 3).

### Per-Protocol Analysis

**Figure 4.**
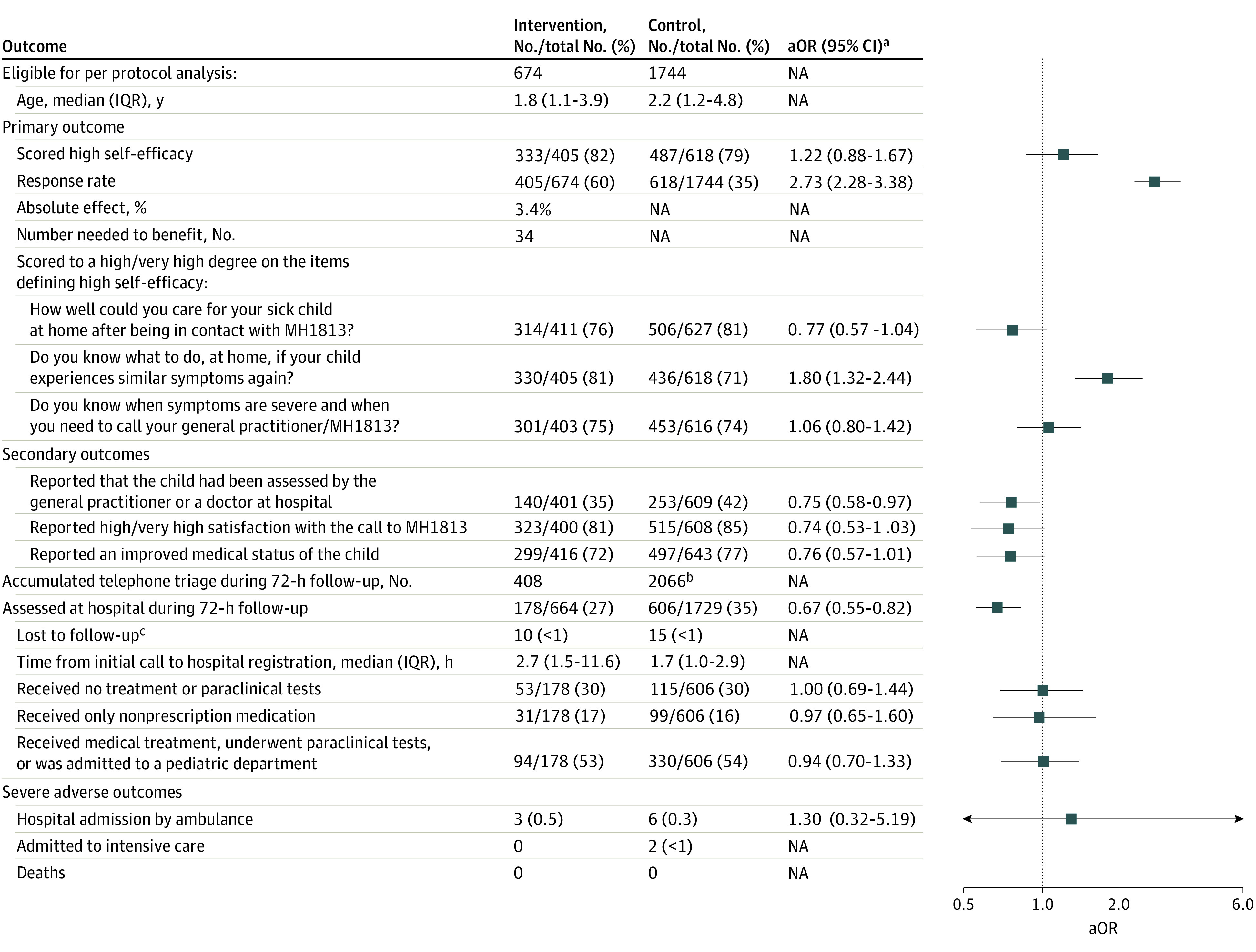
Per-Protocol Subanalysis: Primary, Secondary, and Adverse Outcomes aOR indicates adjusted odds ratio; MH1813, Medical Helpline 1813; NA, not applicable. ^a^Adjusted for categorical age groups (0.5-1.5, 1.6-3.9, and 4.0-11.9 years) using generalized linear model, Rstudio stats package. ^b^Includes the 1744 telephone triage received during the initial call. ^c^Lost to follow-up was caused by incorrect civil registration number.

The per-protocol (PP) analysis included 29% of caregivers (674 of 2307 participants) from the intervention group who viewed 1 or more video tutorials and 73% of caregivers (1744 of 2379 participants) in the control group who received standard telephone triage by a nurse or physician during the initial call. Children assessed at the hospital with an injury were excluded (Figure 4). The median child age was 1.8 (1.1-3.9) years in the intervention group vs 2.2 (1.2-4.8) years in the control group (Figure 4). In total, 82% of caregivers (333 of 405) from the intervention group reported a high self-efficacy vs 79% (487 of 618) in the control group (aOR 1.22; 95% CI, 0.88-1.67). More caregivers in the intervention group reported “to a high/very high degree” to the item “Do you know what to do, at home, if your child experiences similar symptoms again?*”* (81% vs 71%; aOR, 1.80; 95% CI, 1.32-2.44) (Figure 4).

Fewer caregivers in the intervention group reported that their child had been assessed by a family physician or a physician at the hospital (35% vs 42%; aOR, 0.75; 95% CI, 0.58-0.97). Equivalently, fewer children were assessed at the hospital in the intervention group (27% vs 35%; aOR, 0.67; 95% CI, 0.55-0.82).

The remaining findings were similar to the ITT analysis. Details on survey response, telephone triage, and hospital assessments are found in eTable 7, eTable 8, eTable 9, and eTable 10 in Supplement 2.

## Discussion

To our knowledge, this is the first study to examine the efficacy and safety of tutorial videos in empowering caregivers and reducing health care utilization for acutely ill children. Significantly more caregivers in the intervention group who received video tutorials reported high self-efficacy and had reduced health care utilization compared with controls, who received telephone triage. Although not reaching the expected 5% increase in self-efficacy, the 4.4% increase can be considered acceptable as the intervention also reduced health care utilization without increasing adverse outcomes. The impact of the intervention on the individual self-efficacy items appeared to differ, suggesting that the videos were not effective in helping parents in the practical challenges they were encountering with their ill child, but were beneficial in more general terms, potentially empowering caregivers in future situations.

Previous studies^16,19,20^ have investigated empowering caregivers in caring for acutely ill children with mixed results. One study^15^ found increased empowerment, using a 1.5-hour to 4-hour educational session, while another study^17^ reported a 58% decrease in visits to health care facilities using a combination of training programs, a low-literacy self-help book, and consistent follow-up. A multicenter, randomized clinical trial^19^ improved understanding and management of fever using a simple information leaflet during visits to health care facilities. Two randomized clinical trials^14,18^ investigated the effect of written health information during consultations. One trial^14^ found reduced reconsultations and antibiotic use within the same illness episode, while the other trial^17^ showed no impact regarding the use of health services. However, these interventions were not feasible for widespread distribution. Smartphone applications designed for caregivers that provide health information and allow them to score the severity of symptoms have been studied.^29^ However, these applications have shown low usability and raised concern about potentially overtriaging children with mild symptoms to hospital assessment to avoid the risk of overlooking severe illness.^21^

Our study had a unique setup, as the intervention preceded a medical consultation and included data on caregivers’ reported self-efficacy and health care utilization through telephone triage, hospital medical records, and self-reported visits to the general practitioner. Moreover, the video tutorials were feasible for widespread online distribution.

Our findings revealed that approximately one-third of children assessed at the hospital had mild symptoms and did not require treatment or tests, while one-fifth received nonprescription medicine (Figure 2). The high number of children visiting emergency departments for minor illnesses emphasizes the need for improved self-care information.^6,7,8,11,30^ Caregivers have expressed a demand for quality health information,^31^ and institutes such as the American Institute of Medicine, Emergency Medical Services for Children program, and Nuffield Trust have made calls for research on health care utilization.^32,33,34^ Our study suggests that video tutorials can be an effective tool to reduce hospital assessments in children. The great majority of adults in the Western world own a smartphone,^13^ and caregivers often search for information online before seeking medical advice.^35,36^ Considering the high prevalence of hospital assessments in children globally,^6,9,10^ even interventions with modest impact can hold considerable value. Innovative health information such as video tutorials may serve as an important tool to increase health literacy and reduce health care utilization for nonserious, self-limiting illnesses.

### Limitations

This study had certain limitations, including a low participation rate and low viewership of the video tutorials. The intervention group showed increased self-efficacy, but only a third watched the video tutorials. Caregivers in the intervention group who did not watch the video tutorials or made repeated triage calls may have sought advice on home management elsewhere.

The low participation rate affects generalizability, although children of nonparticipating caregivers had the same hospital referral ratio, indicating similar symptom severity. Providing video tutorials alone as intervention assumed they could address caregivers’ worries. However, low participation and viewership suggest caregiver concerns hindered motivation to participate.

Additionally, missing data on self-efficacy limited the analysis, and imputation was not feasible. Surveys carry social desirability bias, but randomization and neutral response options (eg, text message surveys) enable meaningful comparisons. However, the lack of participant blinding introduces response bias. Combining the 3 self-efficacy items into a dichotomized outcome also posed a limitation, and aggregating questions into an ordinal score or analyzing each question separately (given the questionnaire’s brevity) could have enhanced statistical power. Additionally, data were unavailable on parental age, parental socioeconomic status, and the number of siblings, which could impact health care utilization.

Although promising for caregiver empowerment, this study does not reflect real-world conditions due to low participation rate, and the user base might differ from those who participated in the study. Further studies with different setups are needed to assess the effectiveness of video tutorials in empowering caregivers and reducing health care costs, as well as to assess other patient groups.

## Conclusion

Our study found that offering caregivers video tutorials about symptoms in acutely ill children significantly increased caregivers’ self-efficacy and reduced telephone triage, with no increase in adverse outcomes in children. Watching video tutorials also reduced hospital assessments. Good-quality video health care information holds promise for reducing health care utilization in children with mild symptoms.

## Notes

Supplement 1.
Trial Protocol

Supplement 2. **eFigure. **Flow Diagram of Trial Design Including Randomization, Intervention, and Data Collection **eTable 1. **Baseline Characteristics and Response During Telephone Triage at the Medical Helpline 1813 of the Control Group and Those Who Declined to Participate **eTable 2. **Survey Response **eTable 3. **Primary Outcome: Number of Caregivers Who Scored a High Self-Efficacy **eTable 4. **Characteristics of Self-Efficacy Responders and Nonresponders and Outcomes Observed During the 72-Hour Follow-Up Period **eTable 5.** Telephone Triage at the MH1813 During the 72-Hour Follow-Up Period **eTable 6. **Examinations and Admissions to Hospital During the 72-Hour Follow-Up Period **eTable 7. **Per-Protocol Subanalysis: Survey Response **eTable 8. **Per-Protocol Subanalysis: Telephone Triage at the MH1813 During the 72-Hour Follow-Up Period **eTable 9. **Per-Protocol Subanalysis: Primary Outcome (Self-Efficacy), Examinations and Admissions to Hospital During the 72-Hour Follow-Up Period **eTable 10. **Characteristics of Included vs Excluded From the Per-Protocol Subanalysis and Outcomes Observed During the 72-Hour Follow-Up Period

Supplement 3.
Data Sharing Statement
