# Supplementary material for: Video Tutorials to Empower Caregivers of Ill Children and Reduce Health Care Utilization: A Randomized Clinical Trial
Source: JAMA Netw Open. 2023 Oct 12;6(10):e2336836. doi: 10.1001/jamanetworkopen.2023.36836 (PMC10570874; doi:10.1001/jamanetworkopen.2023.36836)
Supplement: Supplement 1. — Trial Protocol [file jamanetwopen-e2336836-s001.pdf]

Dina Cortes, Stine Lund, Fredrik Folke, Morten Schrøder, Marianne Sjølin Frederiksen, Marie Baastrup, Freddy Lippert, Gorm Greisen.

Videos and simple text to empower parents to handle their sick children

Date
5.11-2019

Correspondence
Dina Cortes
Email: dina.cortes@regionh.dk

Introduction

Is it possible – by using videos and simple action cards about common symptoms in children - to empower parents and citizens to take better care of children with milder symptoms at home and to know when to go to a doctor or hospital? The project will help the parents be empowered so they are more confident about caring for their sick children including to know when it is needed to call for help. Competence in caring for children and safety are important aspects of a good family life.

The project addresses a current challenge in the public health sector since many children are referred to hospitals outside their general practitioners' opening hours, and many of these children exhibit only mild symptoms which require neither treatment nor paraclinical tests, but merely medical parental guidance. The project utilises appropriate means, since most parents value the Internet as an information resource. Thus, the material will meet the users where they are – online on their smartphones.

The nature of the problem: a lot of 'trivial' illness in children

Up to the age of four years, children have from 23 to 80 days of sickness a year, where they couldn´t play normally, most days of sickness for children attending day care (1-4). In average the parents reported that the child had had 3.3 episodes of sickness a year and that each period of sickness lasted next to 7 days (4). It may be scaring for parents when their child gets sick, especially when it is for the first time. In about 50% of these contacts, the parents felt the child needed treatment, but in less than 10% of the cases they felt the child was seriously ill (4). Parents with only one child had most medical calls. For children with one or two siblings, doctor's contact was reduced by one third, when compared to first born children, and for children with of three or more siblings' it was reduced to less than one tenth (4). As the average number of children per Danish women is 1.75 only (5), many children are the first-born child.

A Danish study from the Capital Region, in 2013 – the year before the medical helpline 1813 was introduced - found that 28% of the urgent consultations concerned 2.5% of the child population. These children had at least 4 urgent consultations yearly (6). Acute bronchitis was the most frequent diagnosis among these children, which were young (median age of 2.9 years), compared with all the children with an urgent contact (median age of 7.3 years) (6). Moreover, children with young mothers and children with immigrant parents had 37% and 28% increased risk for frequent contact (6), respectively.

A Danish study from the Capital Region, from 2014, the first year of the medical helpline 1813, showed that most calls concerned 0-1-year-old children who seldom exhibited serious conditions (7). The author interpreted the results as if the parents were primarily calling 1813 to get reassurance about the child's condition. When the children got older, the frequency of calls decreased, but in turn, they concerned more serious conditions (7).

Each year there are approximately 190,000 calls to the medical helpline 1813 regarding sick children younger than 12 years (injuries excluded) (8). These children are not so ill that the parents call the emergency helpline 112, but the parents need to contact the health care system outside their general practitioners' opening hours. After contact with the parents, the medical helpline 1813's health professionals may either: 1) refer the child to a paediatric department, 2) refer the

child to assessment in a paediatric urgent care centre (Børnelægevagten), 3) guide the parents to self-care at home, or 4) advised the child to their general practitioners (GP) the next workday.

In 2018, there were 192,000 calls about sick children (injuries excluded) (8). Of these calls, 57% children stayed at home the day the parents called 1813 (31% were guided in self-care and 26% were advised to their GPs the next workday), 40% were referred to the hospital (30% were referred to a paediatric urgent care centre and 10% were referred directly to a paediatric department) and 3% were referred to other options (8).

Many sick children, who are referred to a hospital have mild symptoms which do neither require treatment nor paraclinical tests, but merely parental medical guidance. We estimate it is about 40% of the cases. These consultations can be experienced as unnecessary and inappropriate for the sick child and its parents. Moreover, the visits are expensive for the health system, and the medical staff's attention and time may be directed on the not-so-sick children, so there may be less time for the sicker children referred to a hospital.

A report of the general Danish Emergency Medical Service and the Copenhagen Emergency Service in 2016 showed that children aged 0-4 had the highest number of contacts to the emergency services outside their GPs' opening hours; the number of telephone contacts ranged from 1375 to 1775 per 1000 children. Equivalently, children aged 0-4 years had the highest number of consultations. The number ranged from 475 to 750 per 1000 children, and in Copenhagen it was 500 per 1000 children (9). In Copenhagen 400 per 1000 children aged 0-4 years were referred to the paediatric urgent care centre, like the number in the rest of Denmark (9).

The Internet as a tool to provide an appropriate part of the response to parents' needs

The Internet plays a major role in the daily lives of today's parent, with next to 90% of parent's daily accessing the Internet via their smartphone device (10). It has been reported that parents highly valued the Internet as an information resource in regard to their infant (10-12). They preferred that type of media for information, compared to books and brochures distributed by the hospital from a neonatal intensive care, or a department of Paediatric Surgery (10,11), but a little less than a brochure from a Department of Paediatric otolaryngology (12). However, a high frequency of parents turns to the Internet for medical information (12-15).

There is a concern regarding the quality of the information being available on the Internet (15-16). Reviews conducted in child health related areas, have indicated that quality of health information varies significantly and internet searches may reveal information that is not evidence-based, or may even be inconsistent with international treatment recommendations (16-18). Consequently, there is a need to develop easily accessible evidence-based online information for parents (19).

In Denmark, there is the official homepage https://www.sundhed.dk/, which allows every citizen to assess their own health data from the public health services. Moreover, it includes a homepage called 'patient's handbook' https://www.sundhed.dk/borger/patienthaandbogen/, which offers information about diseases, symptoms, examinations and treatment and help to self-care. However, it is not an app for a smartphone, it may include too much information, it is based on diagnoses and not symptoms, and it is written which results in difficulties for quite many parents. Moreover, the information is not specific for concerns about sick children. Therefore, the

current 'online guidance' is likely too difficult to handle for parents with an acutely sick child. The Danish Committee for Health Education is a non-profit non-governmental organization with close working relations with public authorities like the Ministry of Health, the National Board of Health and private organizations in the health field. Recently, it has developed three apps: "Pregnant", "Father" and "My baby", but they include limited information and advice regarding sick children.

Is it not easy to create a successful eHealth program for empowerment of parents. In India, a large-scale telemedicine program on childhood diarrhoea and pneumonia was not as successful (20), but in Spain a 2-week online educational program covering major topics in food allergy management was successful for parents with children having food allergy (21).

The World Health Organization (WHO) European Regional Office included empowerment and patient-centered practice as key elements in its Health 2020 report (22), a follow-up on previous WHO study on the effectiveness of empowerment to improve health (23). The earlier WHO research identified empowerment as an essential public health strategy but also called for the ongoing refinement of measures to evaluate empowerment (23). Improved definition of patient empowerment and measures to comprehensively evaluate the concept remain (24,25,26), and this is clearly a need for eHealth science as well (27).

Our new material and workflow

There appears to be a case for helping parents with sick children who often are inexperienced and have little access to advice from family or other parents with more experience. In specific, there seems to be a case for empowering parents with regard to their handling of mildly sick children; a case for more self-efficacy. We want to empower these parents to handle mild and common symptoms in children at home by providing easily understood information. We want to focus on symptoms instead of diagnoses as these are the problems of the children. Moreover, we want to tell the parents about danger signs, so they know when they must contact the medical helpline 1813 or a doctor. This means that we want to empower the parents by increasing their self-efficacy.

Study objective

We will produce 8 videos each lasting about 2.5 minutes, about the following common symptoms: 1) vomiting or diarrhea, 2) difficulties in breathing, 3) fever, 4) abdominal pain, 5) sore throat, 6) a red eye, 7) pain in the ears, and 8) exanthemas. The videos will be accompanied by text, action cards, and be accessible on a smartphone. In the beginning the material will be in Danish only, followed in time by materials also in English and Arabic in order to address the increasing demand for languages other than Danish. The material will be developed in collaboration between paediatricians (medical content) and professional film/app-makers (creative content) and will be approved by the Danish Society of Paediatrics and other relevant scientific societies. We want to study the clinical value of this material before we launch the campaign to the public.

The study objective is to investigate if it is possible by using videos and simple action cards about common symptoms in children - to empower parents and citizens to take better care of sick children with milder symptoms at home including to know when they must call for medical help. The corresponding research question is: Is it possible, by videos and simple action cards about eight common symptoms, to increase the number of parents with high self-efficacy in management of the sick child?

Material and methods

Set up

A prospective randomized controlled trial taking place in the Capital Region of Copenhagen including parents who call the medical helpline 1813 abut a sick child aged from 6 months to 11.9 years. Excluded are parents who are not able to speak Danish, who have no Danish telephone number or have no smartphone, children with an injury and those who have participated in the study within the last 72 hours.

The parents who call 1813 will be offered to participate in the study. If they accept, they will be randomized 1:1 to the intervention group (randomized to the new material composed of 8 videos and corresponding smart cards) or to the control group (randomized to current standard care). If the family is randomized to the new material it will be possible to use this material within the next 72 hours after the initial contact to 1813.

The next day in the month at 12 a.m. (at least 12 hours after the call) the participants in the study will receive a text-message with 6 questions, for these questions see Appendix 1. If the parents do not answer the text-message they will get the text-message the following day at 12 a.m.

All case reports of the included children at the hospitals will be reviewed within 3 to 8 days after the call to register the number of children who went to departments of Paediatrics, the diagnosis, if the children had examinations or treatment, if the children were send home from the hospital or admitted to the departments of Paediatrics, the duration of hospitalization, intensive care admittance and mortality within 72 hours after the initial call.

At the Copenhagen Emergency Services the included patients in the study will be identified, and it will be noted the number and the time of further contacts to the medical helpline within the next 72 hours, and if the child was referred to hospital.

Moreover, for every included parent and child (family) it will be noted the number and time the family used the new material, and which kind of the material was used and for how long time.

To investigate if bias was present in the study we want to study: age, sex, if the patients were referred to hospital, admitted to hospital, the duration of hospitalization, intensive care admittance and mortality within 72 hours after the initial call. We also want to study: age, sex, if the patients were referred to hospital for the patients who accepted to participate in the study versus those who did not accept to participate.

Aim of the study

Did the new material about sick children result in high parental self-efficacy among the parents who were allowed to watch the material?
Did new material about sick children result in less children examined by a doctor and more satisfied parents?

Primary outcome

Data of the electronic questionnaire from the parents;

High parental self-efficacy defined as parents who answered" i meget høj grad" or "i høj grad" to at least two of the three questions:

”Hvor godt kunne du passe dit syge barn hjemme, efter du søgte hjælp ved at ringe 1813?”, ”Ved du hvad du kan gøre hjemme, hvis dit barn får samme symptomer en anden gang? and ”Ved du, hvornår det er så alvorligt, at du skal kontakte læge eller 1813, hvis dit barn får samme symptomer en anden gang?”

Secondary outcomes

Data of the electronic questionnaire from the parents;

- Use of professional consultations: Yes to the question ”Har en læge/sygeplejerske undersøgt dit barn under dette sygdomsforløb?” (både egen læge og læge på hospital).
- Satisfied with the contact to the acute telephone 1813: Yes (svar ”i meget høj grad” og ”i høj grad”) to the question “Er du overordnet tilfreds med den hjælp du fik, da du kontaktede 1813?”

Exploratory outcomes

Data of the electronic questionnaire from the parents;

- The medical status of the sick child “Hvordan har dit barn det nu, sammenholdt med da du kontaktede 1813?”

Data from the Copenhagen Emergency Services;

- The number of telephone contacts to 1813, the age and the sex of the child.
- The number of parents who called 1813 within the next 72 hours after the initial call.
- The number of patients, who were referred to a hospital within the next 72 hours after the initial call.

Data of the calls from the videos and action cards;

- The number of times the 8 videos and the eight action cards were used within the next 72 hours after initial contact to 1813.
- The duration and types of videos and action cards the parents watched within the next 72 hours.

Data from the case reports from Epic (Sundhedsplatformen);

- The number of patients, who went to a hospital within the next 72 hours after the initial contact to 1813, and the time of arrival.
- The number and diagnoses of patients who did neither get treatment nor paraclinical tests, but merely parental medical at hospital.
- The number and diagnoses of patients who went home from the hospital with treatment for example antibiotics or painkillers.
- The number of number and diagnoses of sick children who were inpatients in less than 12 hours, more than 12 hours, more than 24 hours in the following 72 hours or went to the intensive care unit.

Automatic speak when the parents call 1813 and the SMS-questions the next day

Please notice **Appendix 1**, in Danish, for specific information.

Safety measures: The paediatric departments in the Capital Region will be made aware of the study. The case reports of the included children at the hospitals will be reviewed within 3 to 8 days after the call. This will primarily be done by the ph.d. student doctor, who is blinded for the information of the study group (intervention versus control group). We will register the number of children who went to departments of Paediatrics, the diagnosis, the duration of hospitalization, intensive care admittance and mortality within 72 hours after the call. It will also be noted, if there are cases of unexpected serious adverse events, near-misses, death, and if it looked as if the child went to hospital later than optimal based on parental delay. In these cases, this information will be presented for a **Data Safety Committee** and it will be revealed if the child was randomized to the intervention or to the control group. The **Data Safety Committee** will advise the study group on continuation, modification, or stopping the trial.

Sample size

We want to be able to detect if the intervention with videos and action cards increases the proportions of parents who expressed high self-efficacy by 5%. We do not know this actual proportion. If it is actual 50%, we want to detect an increase to 55%. We want a p-value of 0.05 and a probability (power) of 80%.
It will require data from about 3300 text-messages (SMSs) in total, randomized in two equal groups (http://www.openepi.com/SampleSize/SSCohort.htm).

If the baseline is at a higher level, for example 70% then we want an to be able to detect an increase to 75%, and with the same p-value and power we then require totally 2520 SMSs.

There are about 160.000 calls concern children of at least 6 months of age to the medical helpline 1813 (18).

If we suggest that 25% of the parents to the sick children agree to be randomized, and that 40% of the included parents answers the text-message the next day, then we can include (160,000 x 0.25)/365 = about 110 children a day, and we will get about 44 text-messages a day. Then we need 2520/44 - 3300/44 = 57- 75 days to include enough patients. It is less than 3 months.

In the data from the Copenhagen Emergency Services, and data from the safety measures we will not have loss to follow up, since data from the Copenhagen Emergency Medical Services and from all four Departments of Paediatrics in the Capital Region are collected routinely.

In the data of the calls from the cases we also suggest that all contacts are safety stored wo we will have no loss to follow-up.

We suggest we have data to answer our research question within 3 months, and the study will be stopped after 6 months.

Stop of the study before an answer our research question

In cases of serious adverse events the study group will consider stopping the study. This will also be the case if the satisfaction with the contact to the acute telephone 1813 drops to a very low level.

Statistics

The primary analysis will use Chi-square and a two-sided level of significance of $p < 0.05$ for the primary outcome and a $p<0.025$ for the two secondary outcomes. All other outcomes are exploratory and the statistical methods will be adapted as appropriate. The results from these exploratory analyses will not but used to claim clinical efficacy but used to design new studies. This is an important aspect since new studies can be rapidly designed and performed once the project set-up is in place.

The dataset will be rich and allow exploration of the effect of individual videos, as well as subgroup analyses.

We believe that our set up is robust, as the sick children are included based on electronic equipment, not adding extra work on the medical personnel, the operators. The follow up to the parents by text message the next day is also based on electronic equipment and the follow-up in the medical case reports after 72 hours, for safety reasons, is based on easily available information, if we have the relevant permits for this.

Ethics and transparency

The parents are informed about the study by an automatic voice in the phone and if they press 1, the value for accept, it is taken as their informed consent. The project will be described in short, like this protocol resumé in Danish, at the webpage of the Copenhagen Emergency Service, and it will be possible to link to the study at https://clinicaltrials.gov/.

The telephone call will be recorded in the Emergency Medical Services system, based on the actual regular routine. Before initiating the study, the protocol will be submitted to the ethics committee of the Capital Region in Denmark, which may consider it outside their mandate. In this case the project will be submitted to ”Videncenter for dataanmeldelse, Rigshospitalet”.

In the project the data will be completely anonymized and it is in no way possible to link data with actual people.

The study will be registered at https://clinicaltrials.gov/ before enrolment of the first patient.

After the first 400 SMS-answers, we will perform an interim analysis to study the frequency of acceptance, and the frequency of high self-efficacy, as well as how the videos have worked technically. Moreover, we will study if the number of SMS-answers from the two groups of families, those randomized to intervention and those randomized to control, are about the same size.

After the first 1000 SMS-answers, we will also make an interim analysis to study the effect of the videos and the action cards. This will also include an examination of a significant difference is exhibited between the number of children staying at home the day the parents call 1813 after having the possibility to watch the videos and read the action cards.

Collaborators

The project will be done in cooperation between the project group of “Børnelægernes Børnetips”, the Copenhagen Emergency Medical Services, the Headquarter at the Capital Region of Denmark and the Faculty of Health and Medical Sciences, University of Copenhagen, Denmark.

The group “Børnelægernes Børnetips” consists of about 15 doctors who are paediatricians or coming-up paediatricians who cooperate with “Children’s Life - see my heart! (Børneliv – se mit hjerte!). A non-profit medical organization run by young doctors www.boerneliv.org, which has experience in doing videos of sick children and in teach children and their teachers in how the body works and emergency help.

Project applicants

Dina Cortes, Professor, MD, DrMedSci, Department of Paediatrics, Copenhagen University Hospital Hvidovre and Faculty of Health and Medical Sciences, University of Copenhagen, and member of the Steering Committee for “Børnelægernes Børnetips”, Denmark. She has experience in using videos of sick children in the teaching and examination of medical students.

Stine Lund, MD, Ph.D., Department of Paediatrics, Copenhagen University Hospital Hvidovre, and member of “Børnelægernes Børnetips”, Denmark. She is behind the Safe Delivery App, which contains animated instructional videos that teach healthcare professionals in low and middle-income countries how to manage birth complications, for example, if the woman starts to bleed after birth or the newborn does not breathe.

Fredrik Folke, Associate professor, MD, Ph.D., Head of research at the Copenhagen Emergency Medical Services and Faculty of Health and Medical Sciences, University of Copenhagen, Denmark. He is also consultant at the Department of Cardiology, Copenhagen University Hospital Gentofte.

Morten Schrøder, MD, Department of Paediatrics, Copenhagen University Hospital Herlev, head of “Børneliv - se mit hjerte!”, and member of the Steering Committee for “Børnelægernes Børnetips” He is in the last part of the education to a pediatrician. He has participated in the public debate on issues at children's departments in Copenhagen, and is also engaged on how to implement ‘play in a hospital setting’ at the new children’s hospital in Copenhagen (BørneRiget at Rigshospitalet).

Marianne Sjølin Frederiksen, MD, Head of the emergency clinic of paediatrics Department of Paediatrics, Copenhagen University Hospital Herlev, and member of the Steering Committee for “Børnelægernes Børnetips”, Denmark. She is also teaching the nurses who work as operators at The Copenhagen Emergency Service.

Marie Baastrup, Head of the medical helpline, RN, Emergency Medical Services Copenhagen. The Emergency Medical Services Copenhagen represents telephone call takers for all patients in the Capital region, outside general practitioners opening hours.

Freddy Lippert, Associate professor, Head, MD, Emergency Medical Services Copenhagen and Faculty of Health and Medical Sciences, University of Copenhagen, Denmark. The Emergency Medical Services Copenhagen represents telephone call takers for all patients in the Capital region, outside general practitioners opening hours.

Gorm Greisen, Professor, MD, DrMedSci, Department of Neonatology, Copenhagen University Hospital Rigshospitalet and Faculty of Health and Medical Sciences, University of Copenhagen,

Denmark. Gorm Greisen has experience in using videos of sick children in the teaching and examination of medical students, and major experience in clinical trials and has been head of the Nation Committee of Ethics.

Time Schedule

- September 2019 – January 2020: Preparing the 8 videos and action cards about the common symptoms; 1) vomiting or diarrhea, 2) difficulties in breathing, 3) fever, 4) abdominal pain, 5) sore throat, 6) a red eye, 7) pain in the ears and 8) exanthema in Danish. Complete the final protocol, obtain ethics approval, and approval of data handling. Register the study at https://clinicaltrials.gov/ before enrolment of the first patient. Preparing the technical system at the medical helpline 1813, for this study. February 2020: Pilot testing of the technical system at the medical helpline 1813.
- March—May/June 2020: Conducting the clinical study. It may be indicated to conduct the study from March to August.
- June 2020 – September 2020: Analysis of results and writing manuscript.
- October 2020: launching of the material, which will be owned and serviced by the Capital Region.

Perspectives and possible yield

In the case of positive effect, it must be decided if the videos and actions cards will be an integrated option at the medical helpline 1813.

Moreover, it may be considered if the material is released to all of Denmark. The material could be accessible at mobile devices phones, iPads, laptops and the material could be placed on a newly developed website for "Børnelægernes Børnetips", on the website for "1813". Parents/citizens can also meet the material on major national websites, Læge-Patient-håndbogen https://www.sundhed.dk/borger/patienthaandbogen/ and https://www.sundhed.dk/sundhedsfaglig/laegehaandbogen/ and Sundhed.dk https://www.sundhed.dk/ and the homepage for Danish Paediatric Society https://www.paediatri.dk

We have contact to all of these, who have accepted to house the material. Moreover, we have contact to the Danish Health Authority. The plan is to empower parents/citizens to take care of children with mild symptoms at home and to know when to go to a doctor/hospital.

We will plan further studies on whether more videos and action cards can be used for children with other symptoms and for children younger than 6 months.

Appendix 1 :

Project information and collection of informed consent

The recorded message will be played when the civil registration number has been validated by the electronical system and the child is aged 0.5-11.9 years.

Pediatricians have developed new videos for parents, so that they can assess and help their sick child – would you like to help us by testing them on your mobile phone?

The purpose of the videos is to guide parents in getting the right help at the right moment and place. If your child has and injury or you are calling from a landline, the films are not relevant for your. Please press 1.

If you agree to receive the videos, a lottery will decide whether you receive videos or are put forward to MH1813. If you choose to participate, you give consent to us reading the medical record if your child is referred to hospital within the next three days. Only legal guardians can participate in the survey.

If you receive the videos, the call will be ended. Feel free to repeat the call to the MH1813 if you want to speak with us about your child after watching the videos.

Press 2 to watch the videos

Press 3 if you wish not to watch the videos. You will be put forward to the MH1813.

Press 9 if you want to hear this recorded message again.

Questionnaire

The questionnaire is received the following day at 12 p.m. If the caregiver does not reply, the questionnaire is sent again on day 2 at 12 p.m. and day 3 at 12 p.m.

Question 1: How is your child now, compared to when you contacted the MH1813

1. *Well*
2. *Better*
3. *Unchanged*
4. *Worse*
5. *Much worse*

Question 2: *How well could you care for your sick child at home, after being in contact with the MH1813?*

1. *To a very high degree*
2. *To a high degree*
3. *To some degree*
4. *To a small degree*
5. *Not at all*

Question 3: "Do you know what to do, at home, if your child experiences similar symptoms again?"

1. *To a very high degree*
2. *To a high degree*
3. *To some degree*
4. *To a small degree*
5. *Not at all*

Question 4: *Do you know when symptoms are severe and when you need to call your General practitioner /MH1813 if these symptoms reappear?*

1. *To a very high degree*
2. *To a high degree*
3. *To some degree*
4. *To a small degree*
5. *Not at all*

Question 5: Have your child undergone an examination by a doctor or nurse at the hospital or general practitioner within 72 hours?"

1. No
2. The child has been seen on video during the telephone triage with MH1813
3. Yes, by the general physician
4. Yes, by a doctor at the hospital

Question 6: Are you overall satisfied with the help you received when contacting the MH1813?

1. *To a very high degree*
2. *To a high degree*
3. *To some degree*
4. *To a small degree*
5. *Not at all*

Thank you for your time

References

1. Lebowitz MD, Cassell EJ, McCarrol J. Health and the urban environment. The incidence and burden of minor illness in a healthy population: familial spread. Am Rev Resp Dis 1972; 106: 824-34.
2. Monto AS, Ullmann B. Acute respiratory illness in an American community. JAMA 1974; 227: 164-9.
3. National Center for Health Statistics. Acute conditions incidence and associated disability. Vital and health statistics. Series 10. Publication No 1000. Washington 1962-78. 1979
4. Uldall P. Spæd- og småbørns almindelige sygelighed – forekomst og sociale konsekvenser. Fadl´s forlag, København 1987, 1-285.
5. Danmarks Statistik. Fødsler https://www.dst.dk/da/Statistik/emner/befolkning-og-valg/foedsler Assessed on 11/03/2019.
6. Region H. Center for Sundhed. Enhed for tværsektoriel Udvikling. Børns brug af regionale sundhedsydelser. https://www.regionh.dk/til-fagfolk/Sundhed/Tvaersektorielt-samarbejde/samarbejdspulje-og-forebyggelse/Documents/rapport-boerns-brug-regionale-sundhedsydelser.pdf Assessed on 11/03/2019.
7. Boisen AS. Reorganization of the emergency care system in the Capital Region of Denmark – An analysis of the interaction between organizational change and user behaviour. Candidate thesis. University of Copenhagen 2015. March 1.
8. Rasmussen mv. Section for data, controlling and IT, Copenhagen Emergency Centre, February 2019.
9. Vinge S, Rasmussen R. Regionale lægevagter og Akuttelefonen 1813 – En kortlægning med fokus på organisering, aktivitet og økonomi. VIVE- Viden til velfærd, Det Nationale Forsknings og Analyse center for Velfærd, 2018. e-ISBN: 978-87-93626-89-8.
10. Orr T, Campbell-Yeo M, Benoit B, Hewitt B, Stinson J, McGrath P. Smartphone and Internet Preferences of Parents. Information Needs and Desired Involvement in Infant Care and Pain Management in the NICU. Advances in Neonatal Care 2017; 17(2): 131-8.
11. Hand F, McDowell DT, Glynn RW, Rowley H, Mortell A. Patterns of Internet use by parents of children attending a pediatric surgical service. Pediatr Surg Int 2013; 29(7): 729e33.
12. Glynn RW, O'Duffy F, O'Dwyer TP, Colreavy MP, Rowley HM. Patterns of Internet and smartphone use by parents of children attending a pediatric otolaryngology service. Int J Pediatr Otorhinolaryngol. 2013; 77(5): 699-702.
13. Thoren EM, Metze B, Bührer C, Garten L. Online support for parents of preterm infants: a qualitative and content analysis of Facebook "preemie" groups. Arch Dis Child Fetal Neonatal Ed. 2013; 98(6): F534-8.
14. De Rouck S, Leys M. Illness trajectory and Internet as a health information and communication channel used by parents of infants admitted to a neonatal intensive care unit. J Adv Nurs. 2013; 69(7): 1489-99.
15. Pehora C, Gajaria N, Stoute M, Fracassa S, Serebale-O'Sullivan R, Matava CT. Are parents getting it right? A Survey of parents' Internet use for children's health care information. Interact J Med Res. 2015; 4(2): e12.
16. Fahy E, Hardikar R, Fox A, Mackay S. Quality of patient health information on the Internet: reviewing a complex and evolving landscape. Australas Med J 2014; 7(1): 24-8.
17. Chung M, Oden RP, Joyner BL, Sims A, Moon RY. Safe infant sleep recommendations on the Internet: let's Google it. J Pediatr. 2012; 161(6): 1080-4.

18. Oncel S, Alvur M. How reliable is the Internet for caregivers on their decision to vaccinate their child against influenza? Results from googling in two languages. Eur J Pediatr. 2013; 172(3): 401-4.
19. O'Kelly F, Nason GJ, Manecksha RP, Cascio S, Quinn FJ, Leonard M, Koyle MA, Farhat W, Leveridge MJ. The effect of social media (#SoMe) on journal impact factor and parental awareness in paediatric urology. J Ped Urol 2017; 13: 513e1e-7
20 Mohanan M, Babiarz KS, Goldhaber-Fiebert JD, Miller G, Vera-Hernández M. Effect of a large-scale social franchising and telemedicine program on childhood diarrhea and pneumonia outcomes in India. Health Aff. 2016 Oct 1;35(10):1800-1809
21 Ruiz-Baqués A, Contreras-Porta J, Marques-Mejías M, Cárdenas Rebollo JM, Capel Torres F, Ariño Pla MN, Zorrozua Santisteban A, Chivato T. Evaluation of an online educational program for parents and caregivers of children with food allergies. J Investig Allergol Clin Immunol. 2018;28(1):37-41).
22 World Health Organization. euro.who. Copenhagen: World Health Organization Regional Office for Europe; 2012. Health 2020: a European policy framework supporting action across government and society for health and well-being http://www.euro.who.int/en/health-topics/health-policy/health-2020-the-european-policy-for-health-and-well-being/about-health-2020 Assessed on 11/03/2019.
23 Wallerstein N. euro.who. Copenhagen: WHO Regional Office for Europe's Health Evidence Network (HEN); 2006. What is the evidence on effectiveness of empowerment to improve health? http://www.euro.who.int/__data/assets/pdf_file/0010/74656/E88086.pdf
24 Bravo P, Edwards A, Barr PJ, Scholl I, Elwyn G, McAllister M, Cochrane Healthcare Quality Research Group, Cardiff University. Conceptualising patient empowerment: a mixed methods study. BMC Health Serv Res 2015 Jul 01; 15: 252.
25 Barr PJ, Scholl I, Bravo P, Faber MJ, Elwyn G, McAllister M. Assessment of patient empowerment--a systematic review of measures. PLoS One 2015; 10(5): e0126553-5.
26 Cerezo PG, Juvé-Udina ME, Delgado-Hito P. Concepts and measures of patient empowerment: a comprehensive review. Rev Esc Enferm USP 2016;50(4):667-674 – 8.
27 Riesling T, Martinez J, Young J, Thorp-Froslie N. Evaluating Patient Empowerment in Association With eHealth Technology: Scoping Review. J Med Internet Res 2017; 19(9): e329.
