# Supplementary material for: Video Tutorials to Empower Caregivers of Ill Children and Reduce Health Care Utilization: A Randomized Clinical Trial
Source: JAMA Netw Open. 2023 Oct 12;6(10):e2336836. doi: 10.1001/jamanetworkopen.2023.36836 (PMC10570874; doi:10.1001/jamanetworkopen.2023.36836)
Supplement: Supplement 2. — eFigure. Flow Diagram of Trial Design Including Randomization, Intervention, and Data Collection eTable 1. Baseline Characteristics and Response During Telephone Triage at the Medical Helpline 1813 of the Control Group and Those Who Declined to Participate eTable 2. Survey Response eTable 3. Primary Outcome: Number of Caregivers Who Scored a High Self-Efficacy eTable 4. Characteristics of Self-Efficacy Responders and Nonresponders and Outcomes Observed During the 72-Hour Follow-Up Period eTable 5. Telephone Triage at the MH1813 During the 72-Hour Follow-Up Period eTable 6. Examinations and Admissions to Hospital During the 72-Hour Follow-Up Period eTable 7. Per-Protocol Subanalysis: Survey Response eTable 8. Per-Protocol Subanalysis: Telephone Triage at the MH1813 During the 72-Hour Follow-Up Period eTable 9. Per-Protocol Subanalysis: Primary Outcome (Self-Efficacy), Examinations and Admissions to Hospital During the 72-Hour Follow-Up Period eTable 10. Characteristics of Included vs Excluded From the Per-Protocol Subanalysis and Outcomes Observed During the 72-Hour Follow-Up Period [file jamanetwopen-e2336836-s002.pdf]

Supplemental Online Content

Borch-Johnsen L, Gren C, Lund S, et al. Video tutorials as a health communication tool to reduce health care utilization: a randomized clinical trial. *JAMA Netw Open*. 2023;6(10):e2336836. doi:10.1001/jamanetworkopen.2023.36836

eFigure. Flow Diagram of Trial Design Including Randomization, Intervention, and Data Collection

eTable 1. Baseline Characteristics and Response During Telephone Triage at the Medical Helpline 1813 of the Control Group and Those Who Declined to Participate

eTable 2. Survey Response

eTable 3. Primary Outcome: Number of Caregivers Who Scored a High Self-Efficacy

eTable 4. Characteristics of Self-Efficacy Responders and Nonresponders and Outcomes Observed During the 72-Hour Follow-Up Period

eTable 5. Telephone Triage at the MH1813 During the 72-Hour Follow-Up Period

eTable 6. Examinations and Admissions to Hospital During the 72-Hour Follow-Up Period

eTable 7. Per-Protocol Subanalysis: Survey Response

eTable 8. Per-Protocol Subanalysis: Telephone Triage at the MH1813 During the 72-Hour Follow-Up Period

eTable 9. Per-Protocol Subanalysis: Primary Outcome (Self-Efficacy), Examinations and Admissions to Hospital During the 72-Hour Follow-Up Period

eTable 10. Characteristics of Included vs Excluded From the Per-Protocol Subanalysis and Outcomes Observed During the 72-Hour Follow-Up Period

This supplemental material has been provided by the authors to give readers additional information about their work.

© 2023 Borch-Johnsen L et al. *JAMA Network Open.*

eFigure1. Flow Diagram of Trial Design Including Randomization, Intervention, and Data Collection

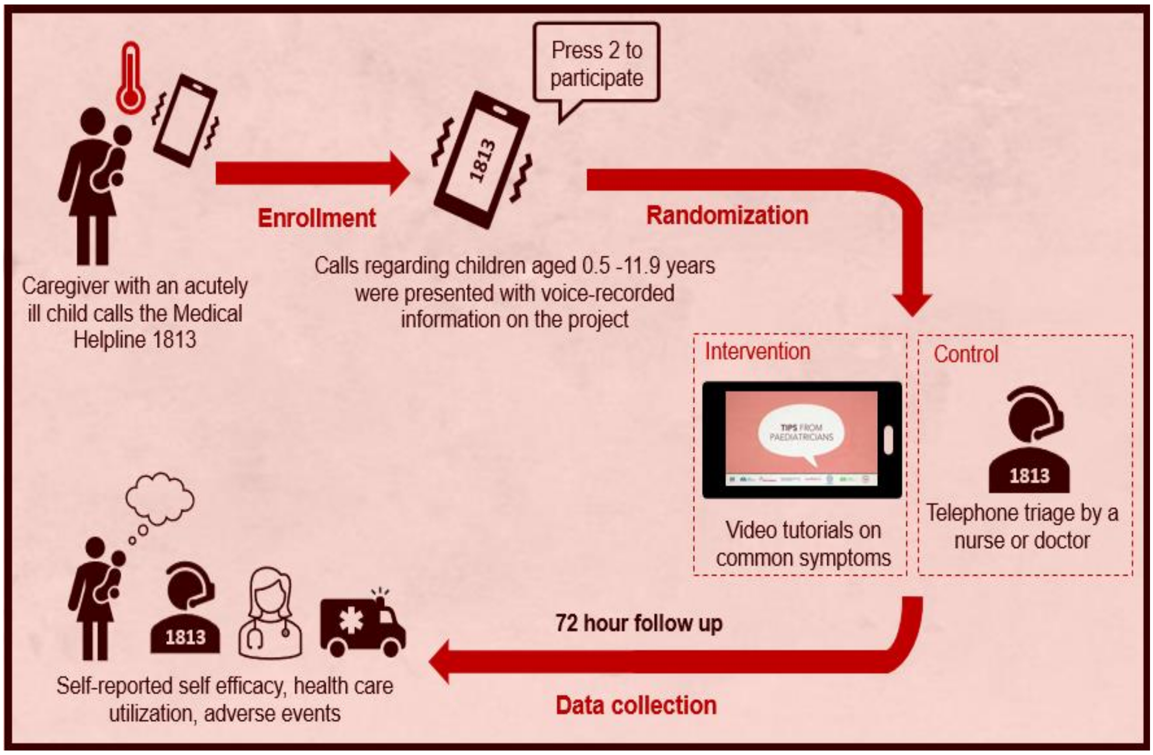

eTable 1. Baseline Characteristics and Response During Telephone Triage at the Medical Helpline 1813 of the Control Group and Those Who Declined to Participate

			cOR[a] [95% CI]	P value
	Control arm[b] (n=2,379)	Declined participation (n=79,940)		
Age/years, median (IQR)	2.4 (1.3-5.0)	2.8 (1.4-6.3)		<.001[c]
Gender, Male	1,266 (53)	42,378 (53)	1.01 [0.93-1.10]	.79
Gender, Female	1,113 (47)	37,562 (47)	0.99 [0.91-1.08]	.84
**Received Telephone Triage at MH1813**	1,920 (81)	69,972 (88)	0.60 [0.55-0.66]	<.001
**Response during telephone triage, No. (%)**				

© 2023 Borch-Johnsen L et al. *JAMA Network Open.*

**Referred to hospital**	653/1,920 (34)	24,012/69,972 (34)	0.97 [0.88-1.07]	.60
Referred for medical evaluation	503/1,920 (26)	16,125/69,972 (23)	1.18 [1.07-1.32]	<.001
Referred with injuries	150/1,920 (8)	7,887/69,972 (11)	0.67 [0.56-0.79]	<.001
Referred to home care with the option to contact the general physician the following day	1,112/1,920 (58)	39,478/69,972 (57)	1.06 [0.96-1.17]	.20
Missing information	155/1,920 (8)	6,482/69,972 (9)	0.86 [0.72-1.01]	.08

Abbreviations: cOR, crude odds ratio; IQR, Interquartile ratio; MH1813, Medical Helpline 1813.
[a] Analyzed with Fishers Exact, Rstudio Epitools package.
[b] Since the intervention arm did not receive telephone triage and subsequent response at the Medical Helpline 1813 during the initial call, the statistical comparison was solely made with the control arm.
[c] Analyzed using the Mann-Whitney-Wilcoxon, Rstudio package.

eTable 2. Survey Response

	**Intervention**						**Control**					
**Item**	**No.**	**Response[a], No. (%)**					**No.**	**Response[a], No. (%)**				
		1	2	3	4	5		1	2	3	4	5
1) How is your child now, compared to when you contacted the MH 1813	791	126 (16)	450 (57)	180 (23)	28 (3)	7 (1)	844	160 (19)	485 (57)	176 (21)	22 (3)	1 (0)

Abbreviations: MH1813, Medical Helpline 1813.
[a] 1 = Well, 2 = Better, 3 = Unchanged, 4 = Worse, 5 = Much worse.

	**Intervention**						**Control**						**P value[b]**
**Item**	**No.**	**Response[a], No. (%)**					**No.**	**Response[a], No. (%)**					
		1	2	3	4	5		1	2	3	4	5	
2) How well could you care for your sick child at home, after being in contact with the MH1813?	774	287 (37)	292 (38)	162 (22)	13 (1)	17 (2)	810	348 (43)	286 (35)	128 (16)	27 (3)	21 (3)	.01
3) Do you know what to do at home, if your child experiences similar symptoms again?	764	249 (33)	334 (44)	149 (19)	17 (2)	15 (2)	794	245 (31)	296 (37)	187 (24)	34 (4)	32 (4)	<.001

4) Do you know when symptoms are severe and when you need to call your General practitioner /MH1813 if these symptoms reappear?	757	248 (33)	331 (44)	153 (20)	17 (2)	8 (1)	787	252 (32)	312 (40)	172 (21)	30 (4)	21 (3)	.11

Abbreviations: MH1813, Medical Helpline 1813.
[a] 1 = To a very high degree, 2 = To a high degree, 3 = To some degree, 4 = To a small degree, 5 = Not at all.
[b] Analyzed using the Mann-Whitney-Wilcoxon, Rstudio package.

	**Intervention**					**Control**				
**Item**	**No.**	**Response[a], No. (%)**				**No.**	**Response[a], No. (%)**			
		1	2	3	4		1	2	3	4
5) Has your child undergone an examination by a doctor or nurse at the hospital or general practitioner within 72 hours?	754	382 (51)	44 (6)	91 (12)	235 (31)	779	365 (47)	55 (7)	91 (12)	268 (34)

Abbreviations: MH1813, Medical Helpline 1813.
[a] 1 = No, 2 = During video triage, 3 = Yes, by the general physician, 4 = Yes, by a doctor at the hospital.

	**Intervention**						**Control**					
**Item**	**No.**	**Response[a], No. (%)**					**No.**	**Response[a], No. (%)**				
		1	2	3	4	5		1	2	3	4	5
6) Are you overall satisfied with the help you received when contacting the MH1813?	749	324 (43)	269 (36)	111 (15)	26 (3)	19 (3)	777	341 (44)	284 (37)	97 (12)	28 (4)	27 (3)

Abbreviations: MH1813, Medical Helpline 1813.
[a] 1 = To a very high degree, 2 = To a high degree, 3 = To some degree, 4 = To a small degree, 5 = Not at all.

eTable 3. Primary Outcome: Number of Caregivers Who Scored a High Self-Efficacy

	**Intervention, No. (%)**	**Control, No. (%)**	**cOR[a] [95% CI]**	**P cOR[a]**
**The no. of caregivers who scored a High Self-efficacy[b]**	**615/764 (80)**	**604/794 (76)**	**1.30 [1.01-1.67]**	**.04**
Age/years, median (IQR)	2 [1.2, 4.6]	2.4 [1.2, 5.0]		.10[c]
Absolute Risk Reduction	4.4%	NA		
Numbers Needed to Benefit, NNB	22.6	NA		

© 2023 Borch-Johnsen L et al. *JAMA Network Open.*

No. of caregivers who completed the questionnaire on self-efficacy	764/2307 (33)	794/2379 (33)	1.01 [0.89-1.45]	.85
No. of caregivers who scored a "High" or "Very High" degree to the items defining self-efficacy:				
Q2: "*How well could you care for your sick child at home, after being in contact with the MH1813?"*,	579/774 (75)	634/810 (78)	0.82 [0.65-1.05]	.11
Q3: "*Do you know what to do, at home, if your child experiences similar symptoms again?"*	583/764 (76)	541/794 (68)	1.51 [1.20-1.90]	< .001
Q4: "*Do you know when symptoms are severe and when you need to call your General practitioner /MH1813?"*	579/757 (76)	564/787 (72)	1.31 [1.02-1.63]	.03

Abbreviations: cOR, crude odds ratio; IQR, Interquartile ratio; MH1813, Medical Helpline 1813.
[a] Analyzed with Fishers Exact, Rstudio Epitools package.
[b] High Self-efficacy was predefined as a score of "To a High or Very high degree" in two out of three of the questions Q2, Q3, or Q4:
[c] Analyzed using the Mann-Whitney-Wilcoxon, Rstudio package.

eTable 4. Characteristics of Self-Efficacy Responders and Nonresponders and Outcomes Observed During the 72-Hour Follow-Up Period

	**Responders**	**Non-responders**	**cOR[a] [95% CI]**	**P cOR[a]**
No. (%)	1,558/4,686 (33)	3,128/4,686 (67)	NA	NA
Intervention arm, no (%)	764/2,307 (33)	1543/2,307 (67)	NA	NA
Control arm, no (%)	794/2,379 (33)	1585/2,379 (67)	NA	NA
Age/years, median (IQR)	2.2 [1.2, 4.8]	2.4 [1.3, 5.2]		.11[b]
Gender, Male	801/1,558 (51)	1,692/3,128 (54)	0.90 [0.79-1.02]	.08[b]
Gender, Female	757/1,558 (49)	1,436/3,128 (46)	1.11 [0.98-1.26]	.08[b]
Phone number registered to, No (%)				
Mother	1003 (64)	1829 (58)		
Father	268 /17)	611 (20)		
Household	73 (5)	137 (4)		
Not traceable	214 (14)	551 (18)		
**Telephone triage at the MH1813 during the 72-hour follow-up period**				
**Caregivers who received telephone triage at the initial call**	680/1,558 (44)	1240/3,128 (40)		

© 2023 Borch-Johnsen L et al. *JAMA Network Open.*

Time from initial call to repeated call to MH1813/hours, median (IQR)	0.5 [0.1, 12.1]	0.3 [0.1, 6.3]		.01[b]
Repeated calls after the initial call:	412	929	NA	NA
0 - 15 min	161 (39)	365 (39)	NA	NA
15.1 - 60 min	43 (11)	114 (12)	NA	NA
1 - 24 hours	128 (31)	294 (32)	NA	NA
24 - 72 hours	80 (19)	156 (17)	NA	NA
Accumulated number of received telephone triage during the 72-hour follow-up period	1092	2169	NA	NA
Accumulated numbers of received telephone triage per child during the 72-hour follow-up period	0.70 (1,092/1,558)	0.69 (2,169/3,128)		.34[c]
**Admissions to hospital**				
No. of children assessed at hospital during the 72-hour follow-up period	590/1,545 (38)	1,135/3,075 (37)	1.06 [0.93-1.20]	.40
Age/years, median (IQR)	2.6 [1.4, 5.6]	2.4 [1.3, 5.7]		.53 [b]
Lost to follow up[d]	13	53		
Time from initial call to hospital arrival/hours, median (IQR)	1.7 [1.0, 3.3]	1.6 [1.0, 3.1]		.08[b]
No. of children who did not receive treatment or paraclinical tests	160 (10)	359 (12)	0.87 [0.71-1.07]	.18
Received treatment/ paraclinical tests/ observation 0–12 hours	402 (26)	721 (23)	1.15 [0.99-1.33]	.06
Admitted to a pediatric department for medical treatment/observation > 12 hours	27(17)	54 (18)	1.00 [0.60-1.62]	1.00
Admitted to intensive care unit	1	1	NA	NA

Abbreviations: IQR, Interquartile ratio; MH1813, Medical Helpline 1813.
[a] Analyzed with Fishers Exact, Rstudio Epitools package.
[b] Analyzed using the Mann-Whitney-Wilcoxon, Rstudio package.
[c] Analyzed using Poisson regression, Rstudio, Stats package.
[d] Lost to follow-up was caused by incorrect Civil Registration Number.
Analysis: The ratio of intervention to controls and the median age show no significant differences between responders and non-responders of the self-efficacy survey. Similarly, the distribution of gender is comparable. Furthermore, the accumulated number of telephone triage received during the 72-hour follow up period does not exhibit any statistically significant differences when analyzed using Poisson regression. The median time from initial call to repeated call to the MH1813 is shorter among non-responders. Additionally, the number of children assessed at the hospital, the median time from initial call to hospital assessment and the type of treatment showed no notable difference between the responders and non-responders.

eTable 5. Telephone Triage at the MH1813 During the 72-Hour Follow-Up Period

	**Intervention, No. (%)**	**Control, No. (%)**	**P value**
Caregivers who received telephone triage at the initial call	**NA**	**1,920 (81)**	NA

© 2023 Borch-Johnsen L et al. *JAMA Network Open.*

Age/years, median (IQR)	2.2 (1.2-4.9)	2 (1.1-4.1)	.08[a]
Time from initial call to first repeated call to MH1813/hours, median (IQR)	0.1 (0.1-8.1)	2.9 (0.4-18.2)	<.001[a]
Repeated calls after the initial call:	887	454	NA
0 - 15 min	451/887 (51)	75/454 (17)	NA
15.1 - 60 min	87/887 (10)	70/454 (15)	NA
1 - 24 hours	222/887 (25)	200/454 (44)	NA
24 - 72 hours	127/887 (14)	109/454 (24)	NA
Accumulated number of received telephone triage during the 72 hour follow-up period	887	2,374[b]	NA
Accumulated numbers of received telephone triage per child during the 72 hour follow-up period	0.38 (887/2,307)	1.00 (2,374/2,379)	<.001[d]

Abbreviations: IQR, Interquartile ratio; MH1813, Medical Helpline 1813.
[a] Analyzed using the Mann-Whitney-Wilcoxon, Rstudio package.
[b] Includes the 1920 telephone triage received during the initial call.
[c] Analyzed using Poisson regression, Rstudio, Stats package.

eTable 6. Examinations and Admissions to Hospital During the 72-Hour Follow-Up Period

	**Intervention, No. (%)**	**Control, No. (%)**	**cOR[a]**	**P cOR**	**aOR[b]**	**P aOR**
Total no. of children assessed at hospital	847/2267 (37)	878/2353 (37)	1.00 [0.88-1.13]	.98	1.00 [0.89-1.13]	.92
Age/years median (IQR)	2.5 (1.3-5.8)	2.7 (1.4-5.4)				
Loss to follow-up[c]	40 (2)	26 (1)				
Time from initial call to hospital arrival/hours, median (IQR)	1.6 (1.0-3.2)	1.7 (1.0-2.9)	NA	>.99		
**Types of examinations /admissions to hospital:**						
No. of children who did not receive treatment or paraclinical tests	251/2,267 (11)	268/2,353 (11)	0.96 [0.80-1.16]	.74	0.97 [0.81-1.16]	.72
Received treatment/ paraclinical tests/ observation 0–12 hours	557/2,267 (25)	566/2,353 (24)	1.03 [0.89-1.18]	.71	1.03 [0.90-1.12]	.63
Received non-prescription medication as only treatment	150 (7)	175 (7)	0.88 [0.70-1.11]	.30	0.87 [0.68-1.10]	.10
Prescribed antibiotics	103 (5)	102 (4)	1.05 [0.79-1.40]	.89	1.06 [0.80-1.40]	.71
Admitted to a pediatric department for medical treatment/observation > 12 hours	39/2,267 (2)	42/2,353 (2)	0.96 [0.60-1.53]	.91	0.97 [0.62-1.50]	.88

Admitted 12-24 hours	15	16	0.97 [0.44-2.11]	> .99	0.94 [0.48-1.98]	.94
Admitted > 24 hours	24	26	0.95 [0.53-1.74]	.89	0.96 [0.55-1.68]	.89
Admitted to intensive care unit[4]	0/2,267 (0)	2/2353 (0.1)	NA	NA	NA	NA
No. of patients who arrived with an ambulance[4]	11/2,267 (0.5)	8/2,353 (0.3)	1.43 [0.45-4.10]	.50	1.43 [0.57-3.57]	.44
Deaths or severe outcomes[d]	0	0	NA	NA	NA	NA
No. of children who did not receive treatment or paraclinical tests among children assessed at hospital	251/847 (30)	268/878 (31)	0.96 [0.78-1.18]	.71	0.98 [0.79-1.20]	.83
No. of children who received non-prescription medication among children assessed at hospital	150/847 (18)	175/878 (20)	0.86 [0.67-1.11]	.24	0.86 [0.68-1.10]	.10
No. of children who received medical treatment, underwent paraclinical tests, or was admitted to a pediatric department among children assessed at hospital	446/847 (53)	435/878 (49)	1.13 [0.93-1.37]	.21	1.11 [0.92-1.34]	.30

Abbreviations: cOR, crude odds ratio; aOR, adjusted odds ratio; IQR, Interquartile ratio; MH1813, Medical Helpline 1813.
[a] Analyzed with Fishers Exact, Rstudio Epitools package.
[b] Adjusted for categorical age groups (0.5-1.5, 1.6-3.9, 4.0-11.9 years) using Generalized linear model, Rstudio stats package.
[c] Lost to follow-up was caused by incorrect Civil Registration Number.
[d] Adverse outcomes.

eTable 7. Per-Protocol Subanalysis: Survey Response

	**Intervention**						**Control**					
**Item**	**No.**	**Response[a], No. (%)**					**No.**	**Response[a], No. (%)**				
		1	2	3	4	5		1	2	3	4	5
1) How is your child now, compared to when you contacted the MH1813	416	58 (14)	241 (58)	102 (25)	13 (3)	2 (0)	643	120 (19)	337 (59)	132 (20)	14 (2)	0 (0)

Abbreviations: MH1813, Medical Helpline 1813.
[a] 1 = Well, 2 = Better, 3 = Unchanged, 4 = Worse, 5 = Much worse.

	**Intervention**						**Control**						**P value[b]**
**Item**	**No.**	**Response[a], No. (%)**					**No.**	**Response[a], No. (%)**					
		1	2	3	4	5		1	2	3	4	5	

2) How well could you care for your sick child at home, after being in contact with the MH1813?	411	147 (36)	167 (40)	86 (21)	4 (1)	14 (2)	627	281 (45)	225 (36)	88 (14)	19 (3)	14 (2)	.008
3) Do you know what to do at home, if your child experiences similar symptoms again?	405	129 (32)	201 (50)	65 (16)	4 (1)	6 (1)	405	197 (32)	239 (39)	136 (22)	26 (4)	20 (3)	.02
4) Do you know when symptoms are severe and when you need to call your General practitioner /MH1813 if these symptoms reappear?	403	119 (30)	182 (45)	90 (22)	9 (2)	3 (1)	616	198 (32)	255 (41)	133 (22)	20 (3)	10 (2)	.84

Abbreviations: MH1813, Medical Helpline 1813.
[a] 1 = To a very high degree, 2 = To a high degree, 3 = To some degree, 4 = To a small degree, 5 = Not at all.
[b] Analyzed using the Mann-Whitney-Wilcoxon, Rstudio package.

	**Intervention**					**Control**				
**Item**	**No.**	**Response[a], No. (%)**				**No.**	**Response[a], No. (%)**			
		1	2	3	4		1	2	3	4
5) Has your child undergone an examination by a doctor or nurse at the hospital or general practitioner within 72 hours?	401	247 (62)	14 (4)	50 (12)	90 (22)	609	307 (50)	49 (8)	65 (11)	188 (31)

Abbreviations: MH1813, Medical Helpline 1813.
[a] 1 = No, 2 = During video triage at the MH1813, 3 = Yes, by the general physician, 4 = Yes, by a doctor at the hospital.

	**Intervention**						**Control**					
**Item**	**No.**	**Response[a], No. (%)**					**No.**	**Response[a], No. (%)**				
		1	2	3	4	5		1	2	3	4	5
6) Are you overall satisfied with the help you received	400	160 (40)	163 (41)	60 (15)	11 (3)	6 (1)	608	283 (47)	232 (38)	70 (11)	13 (2)	10 (2)

© 2023 Borch-Johnsen L et al. *JAMA Network Open.*

when contacting the MH1813?												

Abbreviations: MH1813, Medical Helpline 1813.
[a] 1 = To a very high degree, 2 = To a high degree, 3 = To some degree, 4 = To a small degree, 5 = Not at all.

eTable 8. Per-Protocol Subanalysis: Telephone Triage at the MH1813 During the 72-Hour Follow-Up Period

	**Intervention, No. (%)**	**Control, No. (%)**	**P-value**
No. of caregivers who received telephone triage at the initial call (control group):	NA	1744 (100%)	
Age/years, median (IQR)	1.5 (1.0-2.8)	1.6 (1.0-3.4)	.71[a]
Time from initial call to first repeated call to the MH1813/hours, median (IQR)	0.3 (0.1-7.1)	10.3 (1.9-28.0)	<.001[a]
Total number of repeated calls	408	322	
0-15 min	109 (27)	7 (2)	
15.1 -60 min	32 (8)	29 (9)	
1 -24 hours	222 (54)	200 (62)	
24-72 hours	45 (11)	86 (27)	
Accumulated number of received telephone triage during the 72-hour follow-up period	408	2,066[a]	
Accumulated numbers of received telephone triage per child during the 72-hour follow-up period	0.6 (408/674)	1.2 (2,066/1,744)	<.001[c]

Abbreviations: IQR, Interquartile ratio; MH1813, Medical Helpline 1813.
[a] Includes the 1744 telephone triage received during the initial call.
[b] Analyzed using the Mann-Whitney-Wilcoxon, Rstudio package.
[c] Analyzed using Poisson regression, Rstudio, Stats package.

eTable 9. Per-Protocol Subanalysis: Primary Outcome (Self-Efficacy), Examinations and Admissions to Hospital During the 72-Hour Follow-Up Period

	**Intervention, No. (%)**	**Control, No. (%)**	**cOR[a]**	**P cOR**	**aOR[b]**	**P aOR**
Eligible for per-protocol analysis/	674/2,307 (29%)	1,744/2,379 (73%)				
Age/years, median (IQR)	1.8 (1.1-3.9)	2.2 (1.2-4.8)	NA	.001[1]	NA	.001
**Primary outcome:**						
No. of caregivers who scored a High Self-efficacy	333/405 (82)	487/618 (79)	1.24 [0.89-1.74]	.20	1.22 [0.88-1.67]	.23
Absolute Effect	3.4 %	NA	NA	NA	NA	NA

© 2023 Borch-Johnsen L et al. *JAMA Network Open.*

Numbers Needed to Benefit	34	NA	NA	NA	NA	NA
The response rate on High Self-efficacy, n (%)	405/674 (60)	618/1744 (35)	2.74 [2.28-3.31]	<.001	2.73 [2.28-3.28]	<.001
No. of children examined at hospital during the 72-hour follow-up period	178/664 (27)	606/1729 (35)	0.68 [0.55-0.83]	<.001	0.67 [0.55-0.82]	<.001
Age/years, median (IQR)	1.5 (1.0-3.8)	2.1 (1.3-4.4)	NA	<.001[1]	NA	NA
Time from initial call to hospital arrival, median (IQR)/hours	2.7 (1.5-11.6)	1.7 (1.0-2.9)	NA	<.001[1]	NA	NA
Loss to follow-up[c]	10/674 (2)	15/1744 (1)				
Absolute risk reduction of hospital assessment	8%	NA	NA	NA	NA	NA
Numbers Needed to Benefit to reduce examinations/admission to hospital	12	NA	NA	NA	NA	NA
**Types of examinations/admissions to hospital:**						
Did not receive treatment or paraclinical tests at the hospital	53 (8)	177 (10)	0.76 [0.54- 1.1]	.10	0.73 [0.53-1.01]	.06
Received treatment/ paraclinical tests/observed for 0 – 12 hours	117 (18)	392 (23)	0.73 [0.57-0.92]	.01	0.73 [0.58-0.91]	.01
Received paracetamol as the only treatment	20 (3)	68 (4)	0.75 [0.43- 1.3]	.33	0.72 [0.42-1.2]	.20
Received prescription for antibiotics	34 (5)	82 (5)	1.08 [0.70-1.66]	.67	1.11 [0.73-1.67]	.64
Admitted for medical treatment or observation for more than 12 hours	8 (1)	35 (2)	0.59 [0.23-1.3]	.23	0.61 [0.28-1.33]	.22
Admitted for 12-24 hours	2 (0.3)	13 (0.7)				
Admitted for > 24 hours	6 (1)	22 (1)				
Admitted to intensive care[d]	0	2 (0.1)	NA	NA	NA	NA
No. of patients who arrived with an ambulance[d]	3 (0.5)	6(0.3)	1.30 [0.21-6.12]	.72	1.30 [0.32-5.19]	.72
No. of deaths or severe outcomes[d]	0	0	NA	NA	NA	NA
No. of children who did not receive treatment or paraclinical tests among	53/178 (30)	177/606 (30)	1.03 [0.70-1.50]	.93	1.00 [0.69-1.44]	.98

© 2023 Borch-Johnsen L et al. *JAMA Network Open.*

children assessed at hospital						
No. of children who received non-prescription medication among children assessed at hospital	31/178 (17)	99/606 (16)	1.08 [0.67-1.71]	.73	0.97 [0.65-1.60]	.92
No. of children who received medical treatment, underwent paraclinical tests, or was admitted to a pediatric department among children assessed at hospital	94/178 (53)	330/606 (54)	0.94 [0.70-1.33]	.73	0.99 [0.70-1.39]	.95

Abbreviations: cOR, crude odds ratio; aOR, adjusted odds ratio; IQR, Interquartile ratio; MH1813, Medical Helpline 1813.
[a] Analyzed with Fishers Exact, Rstudio Epitools package.
[b] Adjusted for categorical age groups (0.5-1.5, 1.6-3.9, 4.0-11.9 years) using Generalized Linear Model, Rstudio stats package.
[c] Lost to follow-up was caused by incorrect Civil Registration Number.
[d] Adverse outcomes.

eTable 10. Characteristics of Included vs Excluded From the Per-Protocol Subanalysis and Outcomes Observed During the 72-Hour Follow-Up Period

	**Included**	**Excluded**	**cOR[a] [95% CI]**	**p cOR[a]**
No. (%)	2,418/4,686 (52)	2,268/4,686 (48)	NA	NA
Intervention arm, no (%)	674/2,307 (29)	1,633/2,307 (71)	NA	NA
Control arm, no (%)	1,744/2,379 (73)	635/2,379 (27)	NA	NA
Age/years, median (IQR)	2.1 [1.2, 4.5]	2.6 [1.4, 5.6]		<.001[b]
Gender, Male	1,261/2,418 (52)	1,232/2,268 (54)	0.91 [0.82-1.03]	.14
Gender, Female	1,157/2,418 (48)	1,036/2,268 (46)	1.09 [0.97-1.22]	.14
**Primary outcome:**				
No. of caregivers who scored a High Self-efficacy	820/1023 (80)	399/535 (75)	1.38 [1.06-1.78]	.01
The response rate on High Self-efficacy, n (%)	1,219/2,418 (50)	339/2,268 (15)	4.91 [4.29-5.69]	<.001
**Telephone triage at the MH1813 during the 72-hour follow-up**				

© 2023 Borch-Johnsen L et al. *JAMA Network Open.*

Caregivers who received telephone triage at the initial call	1744	176		
Time from initial call to repeated call to MH1813/hours, median (IQR)	2.6 [0.2, 22.0]	0.1 [0.0, 3.2]		<.001[b]
Repeated calls after the initial call:	504	837		
0 - 15 min	116 (23)	410 (49)		
15.1 - 60 min	61 (12)	96 (11)		
1 - 24 hours	196 (39)	226 (27)		
24 - 72 hours	131 (26)	105 (13)		
Accumulated number of received telephone triage during the 72-hour follow-up period	2248	1013		
Accumulated numbers of received telephone triage per child during the 72-hour follow-up period	0.93 (2,248/2,418)	0.44 (1,013/2,268)		<0.001[c]
**Admissions to hospital**				
No. of children assessed at hospital during the 72-hour follow-up period	784/2,393 (33)	941/2,227 (42)	0.67 [0.59-0.75]	<.001
Age/years median (IQR)	2 [1.2, 4.4]	3.2 [1.6, 6.9]		<.001[b]
Lost to follow-up[d]	25	41		
Time from initial call to hospital arrival/hours, median (IQR)	1.8 [1.1, 3.8]	1.5 [0.9, 2.8]		<.001[b]
No. of children who were assessed at the hospital with an injury	NA	380		
Age/years, median (IQR)	NA	5.5 [3.1, 9.2]		
No. of children who did not receive treatment or paraclinical tests	230/2,393 (29)	289/2,227 (31)	0.71 [0.59-0.86]	<.001
Received treatment/ paraclinical tests/ observation 0–12 hours	509/2,393 (21)	614/2,227 (28)	0.71 [0.62-0.81]	<.001
Admitted to a pediatric department for medical treatment/observation > 12 hours	43 (2)	38 (2)	1.05 [0.66-1.68]	.82
Admitted to intensive care unit[4]	2	0	NA	NA
Admitted with an ambulance	9	10		

Abbreviations: IQR, Interquartile ratio; MH1813, Medical Helpline 1813.
[a] Analyzed with Fishers Exact, Rstudio Epitools package.
[c] Analyzed using Poisson regression, Rstudio, Stats package.
[b] Analyzed using the Mann-Whitney-Wilcoxon, Rstudio package.
[d] Lost to follow-up was caused by incorrect Civil Registration Number.

© 2023 Borch-Johnsen L et al. *JAMA Network Open.*

Analysis: The per-protocol (PP) analysis included 29% (674/2,307) caregivers from the intervention arm who watched video tutorials, and 73% (1,744/2,379) caregivers from the control arm who received standard telephone triage. All children assessed at the hospital with injuries were excluded from the PP analysis since the tutorials did not focus on injuries. The median age in the PP population was 2.1 years, lower than the excluded population (2.6 years), which can be attributed to the children excluded because of injuries. A total of 380 children with injuries, with a median age of 5.5 years, were assessed at the hospital. In the PP analysis, all caregivers in the control arm received telephone triage during their initial call, which makes it unlikely for them to require a repeat call within a 15-minute timeframe. This factor contributes to the observed higher median time from the initial call to the repeated call within the included population (2.6 vs. 0.1 hours). Consequently, the number of accumulated received telephone triage per child was higher for those included in the PP analysis compared to those excluded from the analysis. The 380 children with injuries account for the increased number of children assessed at the hospital in the group in the excluded population. Since most injuries require some form of treatment (nurse assistance, stitches, x-rays, glue, or surgery), this also explains the increased need for treatment. As only fractures requiring surgery are admitted to a pediatric department more than 12 hours, this explains the similar rate of children admitted to a pediatric department.

© 2023 Borch-Johnsen L et al. *JAMA Network Open.*
