# Supplementary material for: Video Tutorials to Empower Caregivers of Ill Children and Reduce Health Care Utilization: A Randomized Clinical Trial
Source: JAMA Netw Open. 2023 Oct 12;6(10):e2336836. doi: 10.1001/jamanetworkopen.2023.36836 (PMC10570874; doi:10.1001/jamanetworkopen.2023.36836)
Supplement: Supplement 3. — Data Sharing Statement [file jamanetwopen-e2336836-s003.pdf]

Data Sharing Statement

Borch-Johnsen. Video Tutorials to Empower Caregivers of Ill Children and Reduce Health Care Utilization. *JAMA Netw Open*. Published October 06, 2023.
doi:10.1001/jamanetworkopen.2023.36836

Data
Data available: Yes
Data types: Deidentified participant data
How to access data: The data can be obtained on request. liv.borch-johnsen.01@regionh.dk
When available: With publication

Supporting Documents
Document types: Statistical/analytic code
How to access documents: The data can be obtained on request.
liv.borch-johnsen.01@regionh.dk
When available: With publication

Additional Information
Who can access the data: researchers whose proposed use of the data has been approved
Types of analyses: For purposes approved by the authors
Mechanisms of data availability: With a signed data access agreement
